# Preparation, Phytochemical Investigation, and Safety Evaluation of Chlorogenic Acid Products from *Eupatorium adenophorum*

**DOI:** 10.3390/molecules22010067

**Published:** 2016-12-31

**Authors:** Boyan Liu, Lili Cao, Lijun Zhang, Xiaofan Yuan, Bing Zhao

**Affiliations:** 1 Division of Biorefinery Engineering, State Key Laboratory of Biochemical Engineering, Institute of Process Engineering, Chinese Academy of Sciences, Beijing 100190, China; lbyone@163.com (B.L.); caoliligood@126.com (L.C.); zhanglijun19861030@126.com (L.Z.); 2 University of Chinese Academy of Sciences, Beijing 100049, China

**Keywords:** *Eupatorium adenophorum*, chlorogenic acid, sesquiterpenes, safety, toxicity

## Abstract

*Eupatorium adenophorum* is widely distributed throughout the world’s tropical and temperate regions. It has become a harmful weed of crops and natural environments. Its leaves contain bioactive compounds such as chlorogenic acid and may be used as feed additives. In this study, chlorogenic acid was extracted and separated from leaves of *E. adenophorum*. Three chlorogenic acid products were prepared with different purities of 6.11%, 22.17%, and 96.03%. Phytochemical analysis demonstrated that the main toxins of sesquiterpenes were almost completely removed in sample preparation procedure. The three products were evaluated for safety via in vitro and in vivo toxicological studies. All the products exhibited no cytotoxic effects at a dose of 400 μg/mL in an in vitro cell viability assay. When administered in vivo at a single dose up to 1.5 g/kg bw, all three products caused no signs or symptoms of toxicity in mice. These results encourage further exploration of extracts from *E. adenophorum* in feed additive application.

## 1. Introduction

*Eupatorium adenophorum* (Crofton weed), an erect perennial weed native to Mexico, is now widely distributed from tropical to temperate regions such as America, Australia, South Africa, Europe, China, and India [1,2]. The leaves of the plant are used to treat fever, diabetes, and inflammation by Nigerian traditional medicine [3]. In India, the plant is used in folk medicine for its antimicrobial, antiseptic, blood coagulating, analgesic, and antipyretic properties [4]. In recent years, phytochemical investigations of *E. adenophorum* have led to the isolation of several bioactive compounds. Polysaccharides from *E. adenophorum* leaves have immunomodulatory properties and a potential prophylactic effect against H5N1 influenza infection [5]. Flavonoids from *E. adenophorum* have antioxidant and antibacterial bioactivities [6,7]. The essential oil extracted from aerial parts of the plant possesses insecticidal and antibacterial properties [4,8].

**Figure 1:**
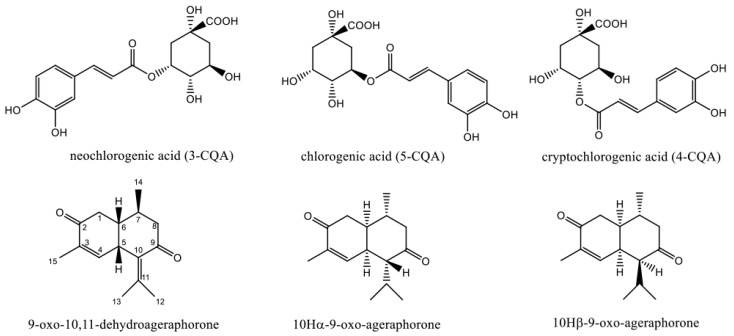
Chemical structures of mono-caffeoylquinic acid (mono-CQAs) and sesquiterpenes in *Eupatorium adenophorum*.

Chlorogenic acid (5-*O*-caffeoylquinic acid, 5-CQA) is one of the major bioactive compounds found abundantly in leaves of *E. adenophorum* [9]. Neochlorogenic acid (3-*O*-caffeoylquinic acid, 3-CQA) and cryptochlorogenic acid (4-*O*-caffeoylquinic acid, 4-CQA) also exist in *E. adenophorum* (Figure 1). They are the three main isomers of mono-caffeoylquinic acid (mono-CQA) in natural plants. They exhibit strong anti-inflammatory [10], anti-bacterium [11], and anti-obesity properties [12]. The products containing different purities of chlorogenic acid extracted from *Eucommia ulmoides* have been used as feed additives for animals [13].

However, *E. adenophorum* is reported as a slight noxious weed [14,15]. Freeze-dried powdered leaves of *E. adenophorum* induced lesions in the liver when fed to mice [16] and rats [17]. Furthermore, the rats administrated with purified extracts from *E. adenophorum* leaves as a diet supplement exhibited hepatotoxicity and cholestasis [18]. The leaves of *E. adenophorum* contain abundant sesquiterpenes [18,19,20,21]. Among them, 9-oxo-10,11-dehydroageraphorone is considered as the main toxin of *E. adenophorum*. It can cause hepatotoxicity in mice [22] and rat [23]. Other cadinene sesquiterpenes such as 10Hα-9-oxo-ageraphorone and 10Hβ-9-oxo-ageraphorone also show toxicity to mice [19] (Figure 1).

As an invasive weed, *E. adenophorum* persists during the dry season and draws attention for its exploitation as a source of forage. Sahoo et al. [24] evaluated the feeding value of *E. adenophorum* in combination with mulberry leaves. Another study suggested that wilting *E. adenophorum* for 24 h could increase its intake by goats. After four weeks, there was virtually no change in goat live weight and no significant difference [25].

Numerous researches have proven that detoxified *E. adenophorum* is an idea feed material [26]. For deep utilization of the invasive weed, it is of considerable interest to know if the extracts from *E. adenophorum* containing chlorogenic acid can be used as feed additives. Thus, in this study, we prepared products from *E. adenophorum* with different purities of chlorogenic acid. Phytochemical evaluation was performed to analyze bioactive components and toxins. Both in vitro and in vivo experiments were carried out to evaluate the safety of the products. It will provide scientific toxin evidence for possible utilization of the weed as a feed additive.

## 2. Results and Discussion

### 2.1. Phytochemical Analysis of the Products

**Figure 2:**
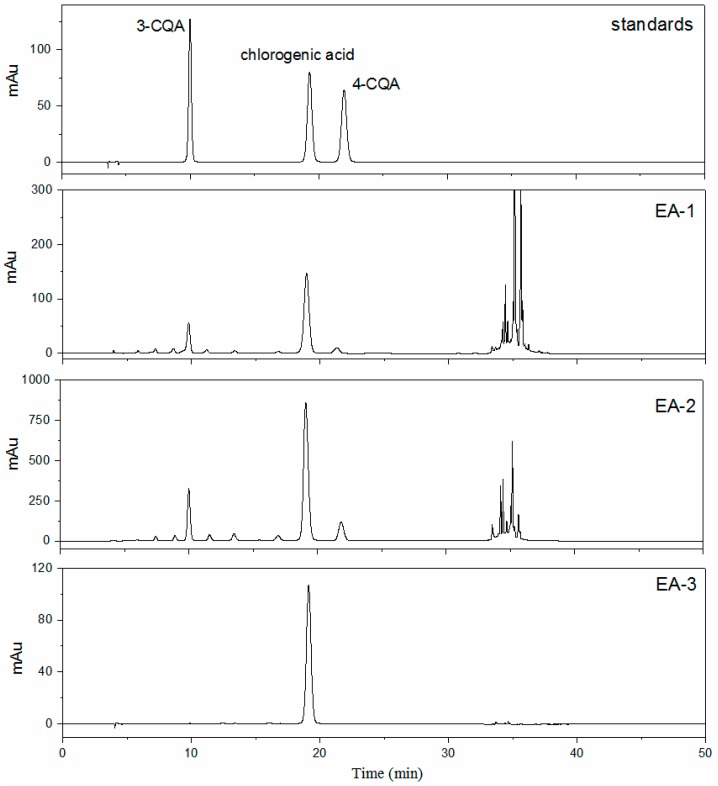
Analytical HPLC chromatograms of mono-CQAs in different products.

A number of compounds with bioactivity have been reported in *E. adenophorum*. Among them, chlorogenic acid, polysaccharides, and flavonoids are the main bioactive components in leaves of *E. adenophorum* [5,6,9,27]. Their contents in different products are presented in Table 1. The phytochemical fingerprint of the crude extract EA-1 (Figure 2) revealed the presence of 3-CQA, chlorogenic acid (5-CQA), and 4-CQA. Chlorogenic acid makes up 6.11% of the product EA-1. After purification by macroporous resin, it reached 22.17% in EA-2. Meanwhile, the contents of 3-CQA and 4-CQA increased from 1.7% and 0.68% in EA-1 to 4.32 and 2.10% in EA-2, respectively. The isomers of mono-CQAs (chlorogenic acid, 3-CQA, and 4-CQA) cannot be separated by macroporous resin, they increased by 3.63-, 2.54-, and 3.09-fold, respectively, in EA-2. Polysaccharides are high polarity substances and can be removed from macroporous resin by water. Thus, total sugars decreased to 4.09% in the product EA-2. Some flavonoids have a similar polarity with the target compound chlorogenic acid, the total flavonoids were also enriched. Due to the existence of chlorogenic acid derivatives and flavonoids, it is rather difficult to get high-purity chlorogenic acid. Different techniques have been used for the separation of high-purity chlorogenic acid from other plants such as high-speed counter-current chromatography [28], pH-zone-refining counter-current chromatography [29], and molecular imprinting [30]. Crystallization is a frequent process step in the manufacturing of active pharmaceutical ingredients. After repeated crystallization by ethyl acetate and water, the purity of chlorogenic acid was above 96%. This is the first report of getting high-purity chlorogenic acid from *E. adenophorum*. The HPLC chromatograms of different products are shown in Figure 2.

**Figure 3:**
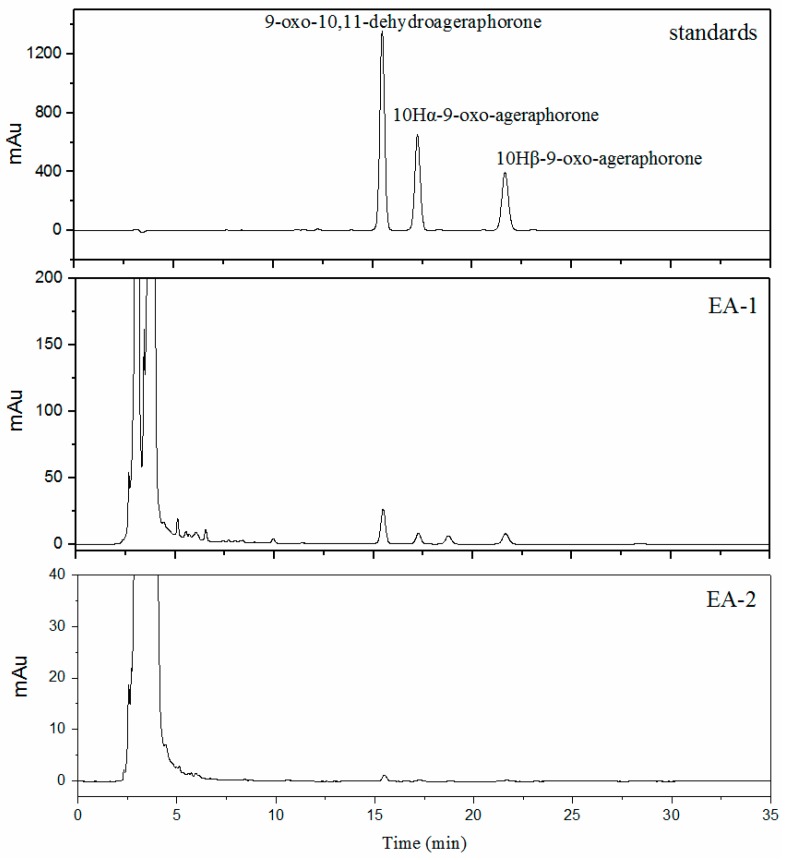
Analytical HPLC chromatograms of sesquiterpenes in different products.

Sesquiterpenes in *E. adenophorum* leaves are known as hazardous materials of hepatotoxicant. However, previous studies have shown that low doses of sesquiterpenes do not adversely affect organs in animals [14,19]. Thus, the contents of the toxins were determined to evaluate the safety of the products. The contents of three main **s**esquiterpenes in different products are summarized in Table 1 and the chromatograms are shown in Figure 3. A significant reduction of **s**esquiterpenes was observed in EA-2 compared to EA-1. The content of 9-oxo-10,11-dehydroageraphorone decreased by about 94%; 10Hα-9-oxo-ageraphorone and 10Hβ-9-oxo-ageraphorone were not detected in EA-2. To reveal if the toxins were within safe levels and evaluate the safety of the products, in vitro and in vivo experiments were needed.

### 2.2. Cytotoxicity Evaluation

Before the in vivo preclinical toxicity test, in vitro tests are useful to assess toxicity in a preliminary way [31]. Human hepatic L02 cells are normal hepatocytes from adult liver tissue. They have been shown to express many specific liver cell functions and could be used in the field of liver toxicity [32,33,34]. HepG2 is a human hepatoma cell line, which is considered as a good model cell line to study xenobiotic metabolism and toxicity to liver [35,36]. Previous reports showed the liver toxicity of *E. adenophorum* to animals [17,18]. The selection of cell lines was based on the main target organs of the potentially adverse or toxic effects of the extracts. We performed a cell counting kit (CCK)-8 assay to assess the effects of different *E. adenophorum* products on cell viability of these two cell lines.

**Figure 4:**
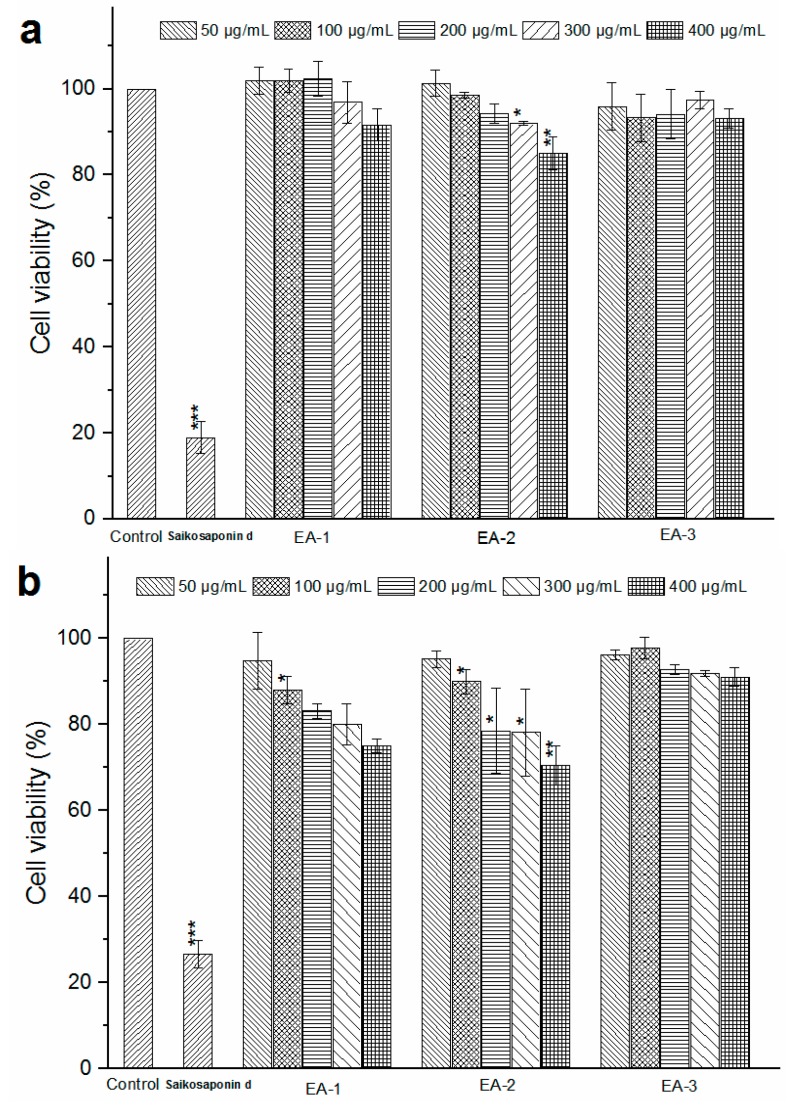
Cell viability of L02 (**a**) and HepG2 (**b**) cells after 24 h incubation with different products at different concentrations, measured by cell counting kit (CCK)-8 assay (Mean ± standard deviation (SD), *n* = 6, * *p* < 0.05, ** *p* < 0.01 and *** *p* < 0.001 compared with the control).

The cytotoxicity of each chlorogenic acid product in L02 and HepG2 cells is shown in Figure 4a,b. Treatment of L02 cells with EA-1 and EA-2 below 200 μg/mL had no significant difference compared to that of the blank control group on the cell survival in 24 h (*p* > 0.05). For HepG2 cells, reductions in cellular viability were observed with increasing concentrations of EA-1 and EA-2 from 50 to 400 μg/mL. However, the half maximal inhibitory concentration (IC_50_) of each product was above 400 μg/mL. Treatment of L02 and HepG2 cells with increasing concentrations of EA-3 (chlorogenic acid) had no effect (*p* > 0.05) on the cell survival up to 24 h. By contrast, saikosdponin d (15 μg/mL) used as a positive standard was highly cytotoxic to L02 and HepG2 cells. A plant extract is generally considered to be not cytotoxic at IC_50_ > 90 μg/mL [37]. Thus, the three products were considered non-cytotoxic.

### 2.3. Acute Oral Toxicity Studies

No deaths or toxic effects such as abnormal behavior were observed at a dose of up to 15 g/kg body weight for all the three products. Their body weights are shown in Table 2. The weights of mice continued to increase. Finally, no treatment-related gross pathological changes were observed in any organs (kidney, liver, lung, spleen, heart, colon, and thymus) of the test animals during necropsy. The results indicate that the products with different purities of chlorogenic acid (EA-1, EA-2, and EA-3) have no toxicity at the doses tested in this work.

Previous studies have found that cadinene sesquiterpenes work as the main toxins of *E. adenophorum*. In a previous investigation, the highest non-fatal oral dose of 9-oxo-10,11-dehydroageraphorone for male mice was found to be 350 mg/kg body weight [22]. Ouyang et al. [14] examined the toxicity of sesquiterpenes from *E. adenophorum* in mice. In an acute study, the median lethal dose (LD_50_) of 9-oxo-10,11-dehydroageraphorone, 9-oxo-agerophorone, and 2-deoxo-2-(acetyloxy)-9-oxo-ageraphorone was 1470, 1470, and 926 mg/kg body weight (bw), respectively, for male mice. In a sub-acute study, a 75 mg/kg dose of 2-deoxo-2-(acetyloxy)-9-oxo-ageraphorone or 9-oxo-agerophorone was found to be approximately or totally safe. Cadinene sesquiterpenes in *E. adenophorum* were regarded as a type of low toxicity botanical component. In our experiment, the single doses of 9-oxo-10,11-dehydroageraphorone, 10Hα-9-oxo-ageraphorone, and 10Hβ-9-oxo-ageraphorone in product EA-1 to mice were calculated to be 60.15, 34.05, and 38.85 mg/kg bw, respectively. This is a safe dose for mice. The oral dose of 9-oxo-10,11-dehydroageraphorone in product EA-2 to mice was calculated to be 3.6 mg/kg bw, and 10Hα-9-oxo-ageraphorone and 10Hβ-9-oxo-ageraphorone were below the detection limit. EA-2 was regarded as a rather safe product.

*E. adenophorum* is widely distributed as an invasive plant, considerable effort has been made to find new and innovative methods for its management. *E. adenophorum* holds promise as a source of feed additives for practical use. The use of *E. adenophorum* leaf extract as a source of feed additive represents an alternative strategy in the comprehensive management of invasive weeds.

## 3. Materials and Methods

### 3.1. Materials and Chemicals

Leaves of *E. adenophorum* were collected from Sichuan Province, China, in June 2014, and provided by Xiyu Biotech Co., Ltd. (Panzhihua, China). Standards of chlorogenic acid, saikosdponin d, and rutin were purchased from the National Institute for the Control of Pharmaceutical and Biological Products (Beijing, China); the purities of all the standards were not less than 96%. Products 3-CQA and 4-CQA were obtained from Chengdu Must Biotechnology Co. Ltd. (Chengdu, China), and the purities were up to 98%. Products 9-oxo-10,11-dehydroageraphorone, 10Hα-9-oxo-ageraphorone, and 10Hβ-9-oxo-ageraphorone were isolated from the leaves of *E. adenophorum* by our group and identified by NMR and mass spectrometry analyses. The data were in agreement with published data [23,38,39,40,41]. The purities were over 98% according to the HPLC determination result. NKA-II macroporous resin was purchased from Bon Adsorber Technology Co., Ltd. (Cangzhou, China).

### 3.2. Preparation of Different Chlorogenic Acid Products from E. adenophorum

The dried leaves of *E. adenophorum* (1 kg) were extracted with 20 L of 60% (*v*/*v*) ethanol at 80 °C for 60 min. The solution was then filtered and the supernatant was evaporated with a rotary evaporator (RE-52A, Shanghai Yarong Biochemical Instrument Factory, Shanghai, China) under reduced pressure at 60 °C. The concentrated solution (solution 1) was dried by a spray dryer (LPG-5, Kaiou Instrument Co., Ltd., Nanjing, China) to get the powder EA-1. The spray drying conditions were as follows: solution concentration, 1.04 g/mL; inlet air temperature, 160 °C; sample flow rate, 2.5 L/h; drying air flow, 20 m^3^/min.

The concentrated solution was then purified by macroporous resin (NKA-II; polar; surface area, 160–200 m^2^/g; average pore diameter, 145–155 Å) based on our reported method [9]. Briefly, the experiment was carried out on a glass column (1000 mm × 70 mm i.d.) and the bed volume (BV) of the resin was 2.0 L. A 6.0 L concentrated solution (solution 1, with chlorogenic acid 4 mg/mL) was subjected to the column at the flow rate of 1 BV/h. The column was washed by 3 BV of deionized water to remove the high polar components. Then the column was desorbed by 3 BV of 40% (*v*/*v*) ethanol at the flow rate of 2 BV/h. The 40% (*v*/*v*) ethanol desorption solution was concentrated and freeze dried (FD-80, Boyikang Instruments Co., Ltd., Beijing, China) for 24 h (−50 °C, 0.04 mbar vacuum pressure) to get powder EA-2.

A 100 g sample of the powder EA-2 was dissolved in 2 L deionized water and then extracted by three successive treatments with 1 L ethyl acetate. The three ethyl acetate phases were pooled and concentrated to 1 L and then extracted with 1 L deionized water three times. The water phases were concentrated to 150 mL and crystallized at 4 °C. The chlorogenic acid crystal was dried in a vacuum oven (DZ-1BC, Teste Instrument Co., Ltd., Tianjin, China) at 60 °C for 4 h in order to obtain the powder EA-3.

### 3.3. Quantitative Analysis of Selected Phytochemical Constituents

#### 3.3.1. HPLC Analysis of Mono-CQAs

The analytical method for the determination of mono-CQAs (3-CQA, chlorogenic acid, and 4-CQA) was taken from a previously published method [9]. The analysis was carried out on an Agilent 1260 series HPLC system (Palo Alto, CA, USA). A Waters XTerra C18 column (4.6 mm × 250 mm, 5 μm) was used, and the temperature was maintained at 30 °C. The mobile phases were 0.2% formic acid aqueous solution (A) and acetonitrile (B). The gradient elution program was as follows: 0–27 min, 8% of B; 27–23 min, 8%–50% of B; 33–40 min, 50%–8% of B; 40–50 min, 8% of B. The detection wavelength was 327 nm, the flow rate was 0.8 mL/min, and the injection volume was 5 μL. The compounds were identified by comparing the retention times with the standards.

#### 3.3.2. HPLC Analysis of Sesquiterpenes

For sesquiterpenes (9-oxo-10,11-dehydroageraphorone, 10Hα-9-oxo-ageraphorone, and 10Hβ-9-oxo-ageraphorone), the detection wavelength was 254 nm and the mobile phase was 40% acetonitrile aqueous solution. Other HPLC conditions were the same as shown in the text Section 3.3.1.

#### 3.3.3. Quantification of Total Sugars Content

Total sugars were quantified by the phenol–sulfuric acid method using d-galactose as standard [42].

#### 3.3.4. Quantification of Total Flavonoid Content

Flavonoids concentration was determined by the colorimetric method [43,44]. A 1 mL sample of diluted solution and 1 mL of 5% (*w*/*v*) NaNO_2_ were mixed for 6 min. Then, 1 mL of 10% AlCl_3_ (*w*/*v*) was added. After 6 min, 10 mL of 1 mol/L NaOH was added. After the solution was left standing for 15 min，the absorbance of the solution was measured at 510 nm using a spectrophotometer (UV-2802, Unico, Dayton, NJ, USA). Rutin was used to construct the standard curve.

### 3.4. Study of Toxicity in Vitro

#### 3.4.1. Cell Culture and Treatment

Human hepatocyte cell line L02 and hepatocellular carcinoma cell line HepG2 were obtained from China Cell Culture Center (Shanghai, China). Cells were grown in Dulbecco’s minimum essential medium (Gibco-BRL, Gaithersburg, MD, USA), containing 10% fetal bovine serum (FBS), 1% penicillin (100 IU/mL), and streptomycin (100 μg/mL), at 37 °C in humidified 5% CO_2_. The medium was changed every 2 days. Cells in the exponential growth phase were used in the experiments.

Two types of cells were treated with the corresponding medium containing different concentrations of the sample. Different products were diluted with the culture medium (0.1% DMSO) into different concentrations (50, 100, 200, 300, and 400 μg/mL).

#### 3.4.2. Cell Viability Assay

Cell viability was determined by a Cell Counting Kit (CCK)-8 (Dojindo, Kumamoto, Japan) assay. Freshly collected L02 and HepG2 cells were seeded in 96-well plates (1 × 10^4^ cells/well) and cultured for 24 h to obtain a monolayer culture. Then, the cells were incubated with fresh media containing various concentrations of samples for 24 h. After incubation, the culture medium was replaced with 100 μL fresh medium and 10 μL CCK-8 solution. The cells were further incubated at 37 °C in humidified 5% CO_2_ for 1 h. The absorbance of the samples was measured at 450 nm using a microplate reader (Multiskan FC, Thermo Scientific, Waltham, MA, USA.). The wells with cell and culture medium (0.1% DMSO) but without samples were considered as normal control, and the wells with only culture medium were considered as blank control. Saikosaponin d (15 μg/mL), a typical liver toxicant, was used as a positive control. The cell viability was calculated by the absorbance divided by the normal control group after subtracting the value of the blank control.

### 3.5. Study of Toxicity In Vivo

#### 3.5.1. Animals

The ICR mice (aged 7–10 weeks, weighing 25–35 g, certificate no. SCXK-(JING)-2011-0012) were provided by The Department of Laboratory Animal Science, Peking University Health Science Center. The animals were housed in stainless steel wire cages with six mice per cage in an air-conditioned room (24 ± 2 °C, relative humidity of 55% ± 10%, an air ventilation frequency of 15 times/h, and a 12 h light/dark cycle). All these animals had free access to food and water and were allowed to acclimatize for 72 h before initiation of the experiments. Animal handling and procedures were performed according to the ethical guidelines of Peking University Health Science Center (Certificate No. SCXK-(JING)-2012-0011).

#### 3.5.2. Acute Oral Toxicity

Thirty male and 30 female ICR mice were randomly divided into three treatment groups, 10 male and 10 female mice for each group. The powder of different products was dissolved in distilled water and administered to the mice via oral gavage in a single dose of 15.0 g/kg body weight (0.2 mL solution/10 g bw, all the products were at the maximal concentration of 0.75 g/mL). The signs of toxic effects and mortality were observed carefully every 0.5 to 1 h after administration on the first day, followed by daily observation for their symptoms for 14 days. The mice were weighed initially and then every 7 days throughout the study. After 14 days, all organs were examined for gross pathological changes.

### 3.6. Statistical Analysis

The statistical significance was determined using a one-way analysis of variance (ANOVA). Results were classified into three significance levels when the *p*-value were < 0.05, 0.01, and 0.001.

## 4. Conclusions

In conclusion, in this paper, three chlorogenic acid products were prepared from *E. adenophorum* with the different purities of 6.11%, 22.17%, and 96.03%. The bioactive components including mono-CQAs, polysaccharides, and flavonoids were determined. The main toxins were almost completely removed in the sample preparation procedure. In order to evaluate the safety of the products and provide data for feed additive application, the toxicity of the products was evaluated by both cell and animal models. Cell culture results revealed that the three products had no obvious negative effect on cell viability when the concentration was increased to 400 μg/mL in the culture medium. The acute toxicity evaluation demonstrated the safety of the three products at a high dose of up to 15 g/kg bw of mice. The results of these studies suggest that extracts from *E. adenophorum* may be safe as feed additives. Chronic toxicity, mutagenicity, carcinogenicity, and teratogenicity studies are desired to further support the safe use of this plant.

## Figures and Tables

**Table 1:** Phytochemical analysis of different products.

Product	3-CQA (%)	Chlorogenic Acid (%)	4-CQA (%)	Total Sugars (%)	Total Flavonoids (%)	9-oxo-10,11-Dehydroageraphorone (mg/g)	10Hα-9-oxo-Ageraphorone (mg/g)	10Hβ-9-oxo-Ageraphorone (mg/g)
EA-1	1.70	6.11	0.68	10.26	15.25	4.01	2.27	2.59
EA-2	4.32	22.17	2.10	4.09	33.73	0.24	–	–
EA-3	– ^1^	96.03	–	–	–	–	–	–

^1^ Under the limit of detection.

**Table 2:** Body weight of mice fed with different *E. adenophorum* products at a dose of 15 g/kg body weight (bw).

Group	Sex	Body Weight (g)			
		Initial	1st Day	7th Day	14th Day
EA-1	Female (*n* = 10)	24.94 ± 0.60	24.53 ± 0.60	25.79 ± 0.83	27.85 ± 1.53
	Male (*n* = 10)	27.54 ± 1.28	27.75 ± 1.43	31.08 ± 2.10	34.98 ± 2.91
EA-2	Female (*n* = 10)	25.58 ± 0.70	24.91 ± 1.01	25.85 ± 0.83	27.79 ± 1.22
	Male (*n* = 10)	26.92 ± 1.49	27.82 ± 1.38	31.08 ± 1.80	33.66 ± 1.94
EA-3	Female (*n* = 10)	26.54 ± 0.92	25.36 ± 0.60	27.83 ± 1.46	30.02 ± 1.68
	Male (*n* = 10)	32.86 ± 1.60	32.73 ± 1.42	36.88 ± 1.75	38.37 ± 2.36

Values are expressed as mean ± SD.
